# asymptoticMK: A Web-Based Tool for the Asymptotic McDonald–Kreitman Test

**DOI:** 10.1534/g3.117.039693

**Published:** 2017-03-24

**Authors:** Benjamin C. Haller, Philipp W. Messer

**Affiliations:** Department of Biological Statistics and Computational Biology, Cornell University, Ithaca, New York 14853

**Keywords:** molecular evolution, positive selection, web service

## Abstract

The McDonald–Kreitman (MK) test is a widely used method for quantifying the role of positive selection in molecular evolution. One key shortcoming of this test lies in its sensitivity to the presence of slightly deleterious mutations, which can severely bias its estimates. An asymptotic version of the MK test was recently introduced that addresses this problem by evaluating polymorphism levels for different mutation frequencies separately, and then extrapolating a function fitted to that data. Here, we present asymptoticMK, a web-based implementation of this asymptotic MK test. Our web service provides a simple R-based interface into which the user can upload the required data (polymorphism and divergence data for the genomic test region and a neutrally evolving reference region). The web service then analyzes the data and provides plots of the test results. This service is free to use, open-source, and available at http://benhaller.com/messerlab/asymptoticMK.html. We provide results from simulations to illustrate the performance and robustness of the asymptoticMK test under a wide range of model parameters.

The extent to which molecular evolution is driven by positive selection, rather than neutral evolutionary processes such as random genetic drift, is one of the central questions of modern evolutionary biology. This question can be studied quantitatively by estimating the parameter *α*, which specifies the fraction of nucleotide substitutions in a given genomic region that were driven to fixation by positive selection (Eyre-Walker 2006). Values of *α* close to one indicate that most substitutions in the region were indeed the result of positive selection, whereas values close to zero indicate neutral evolution.

One of the most widely used approaches for inferring *α* from polymorphism and divergence data is the McDonald-Kreitman (MK) test (McDonald and Kreitman 1991; Eyre-Walker 2006), which compares levels of divergence between a genomic test region and a neutrally evolving reference region with the levels of polymorphism in the two regions. Early applications of the MK test typically focused on nonsynonymous sites in protein-coding regions as the test region, while synonymous sites were used as the neutral reference. However, the approach can also be applied to arbitrary genomic compartments or classes of mutations (Andolfatto 2005).

The original MK test makes several critical assumptions about the nature of the evolutionary process. First, it assumes that the positively selected mutations that ultimately contribute to divergence in the test region go to fixation quickly, such that they do not contribute noticeably to polymorphism levels. Second, it assumes that deleterious mutations in the test region are sufficiently deleterious to be lost quickly, such that they contribute to neither polymorphism nor divergence. Finally, neutral mutations in the test region are assumed to be subject to drift similar to the mutations in the neutral reference region and can therefore contribute to both polymorphism and divergence. Under these assumptions, it holds that

$$\alpha =1-\frac{d_{0}}{d}\frac{p}{p_{0}},$$ (1)

where *d* and *d*_0_ are substitution rates per site in the test region and neutral reference region, respectively, while *p* and *p*_0_ specify the respective levels of polymorphism per site in the two regions (Eyre-Walker 2006). Note that if polymorphism and divergence levels are estimated over the same region, the total number of sites cancels out in the ratios *p*/*d* and *d*_0_/*p*_0_, and one may then simply use the actual counts of observed substitutions (*D* and *D*_0_) and polymorphic sites (*P* and *P*_0_) instead of rates per site (Eyre-Walker 2006).

With the growing availability of genome-level polymorphism and divergence data sets, the MK test has become a popular method for inferring positive selection in various organisms (Fay 2011). Several software tools and web services with implementations of the test have also been developed (Egea *et al.* 2008; Librado and Rozas 2009; Eyre-Walker and Keightley 2009; Stoletzki and Eyre-Walker 2011; Vos *et al.* 2013). The estimates of *α* obtained in these studies range from as high as ∼0.5 for nonsynonymous substitutions in *Drosophila* (Sella *et al.* 2009), to close to zero in organisms such as yeast (Elyashiv *et al.* 2010) or many plants (Gossmann *et al.* 2010). Indeed, estimates of *α* obtained from Equation (1) are often negative, indicating that at least some of the assumptions of the test were likely not met (since negative values of *α* have no biological meaning - estimates of *α* may be negative, but the true value cannot be).

One major problem with the original MK test lies in its assumption that deleterious mutations do not contribute to polymorphism in the test region. This stands in contrast to the frequent observation of weakly deleterious mutations in many organisms, and the fact that such mutations can substantially affect the site frequency spectrum (SFS) of polymorphisms in functional genomic regions (Bustamante *et al.* 2005; Eyre-Walker *et al.* 2006). In the presence of weakly deleterious mutations, the observed level of polymorphism in the test region (*p*) in Equation (1) will overestimate the rate at which neutral polymorphisms are expected to go to fixation in this region, which will bias estimates of *α* downward (providing one possible explanation for the frequent observation of negative *α* estimates).

As one strategy to address this problem, it has been proposed to only consider polymorphisms for which the derived allele is above a certain threshold frequency when estimating *p* and *p*_0_ (Charlesworth and Eyre-Walker 2008). This is because the fraction of weakly deleterious mutations among all polymorphisms should be lower for higher derived- allele frequencies. Ideally, one would wish to set this cutoff high, to minimize the bias due to weakly deleterious mutations; however, the higher this cutoff, the fewer polymorphisms will actually remain in the data set, thus increasing statistical noise. To circumvent this problematic tradeoff, more sophisticated extensions of the original MK test first attempt to infer the actual distribution of fitness effects among new mutations in the test region from the SFS, and then correct fixation probabilities accordingly (Boyko *et al.* 2008; Eyre-Walker and Keightley 2009). Yet these approaches can still suffer from unknown effects of demography or linked selection that are also expected to affect the shape of the SFS. The most sophisticated extensions of the test therefore additionally incorporate basic demographic models to improve their estimates (Keightley and Eyre-Walker 2007; Boyko *et al.* 2008; Eyre-Walker and Keightley 2009), which requires additional (and often uncertain) assumptions about the demographic history of the population of interest.

In contrast to such model-based approaches, a considerably simpler, heuristic approach was recently proposed by Messer and Petrov (2013). This approach generalizes the frequency-cutoff approach described above, without the need to discard polymorphism data. Instead of setting a specific frequency cutoff, it separately estimates *α* for each of a set of discrete mutational frequency classes:

$$\alpha \left(x\right)=1-\frac{d_{0}}{d}\frac{p\left(x\right)}{p_{0\left(x\right)}}.$$ (2)

Here, *p*(*x*) and *p*_0_(*x*) specify the levels of polymorphism per site in the test and reference regions, respectively, considering only those polymorphisms for which the derived allele is present at frequency *x* in the population (estimated from a population sample, for example). In the presence of deleterious mutations, *α*(*x*) will underestimate the true value of *α* for small *x*, yet should converge to the correct value as *x* approaches one (Messer and Petrov 2013). The asymptotic estimate of *α* is then obtained by fitting a function *α*_fit_(*x*) to the empirical *α*(*x*) values and extrapolating this function to *x* = 1:

$$\alpha_{\mathrm{asymptotic}}=\alpha_{\mathrm{fit}}\left(x=1\right).$$ (3)

One key advantage of this approach is that, because *α*(*x*) does not depend on the individual functions *p*(*x*) and *p*_0_(*x*) but only on their ratio, any biases due to demography or linked selection that affect the SFS in the test and reference regions in the same way will effectively cancel out (Messer and Petrov 2013). Another advantage over model-based approaches is that the asymptotic MK approach is much more computationally efficient, as it requires only fitting a simple curve to the data.

In this paper, we present asymptoticMK, a web-based tool for executing the asymptotic MK test quickly and easily in any web browser. After the necessary values are entered, asymptoticMK generates analyses and plots that are directly usable in publications. It is based internally on R, but no knowledge of R is needed to use it, nor does the user of asymptoticMK need to have R installed on their computer. For those who do wish to run the test themselves in R, the necessary code is freely available online. The asymptoticMK service can also be run in an automated fashion at the command line, for bulk analysis in script-based workflows. Finally, we present results from forward genetic simulations to illustrate the performance and robustness of the asymptotic MK test in various scenarios.

## Methods

### Implementation

The asymptoticMK web service is implemented in R (R Development Core Team 2016). It uses the package FastRWeb (Urbanek 2008) to parse HTTP requests and generate responses, and uses the package Rserve (Urbanek 2003) as the lower-level interface that communicates with the web server through the standard CGI mechanism. A version of asymptoticMK that runs in R on the user’s local machine is also provided. Source code and additional resources related to asymptoticMK are posted on GitHub at https://github.com/MesserLab/asymptoticMK.

### Usage

**Figure 1:**
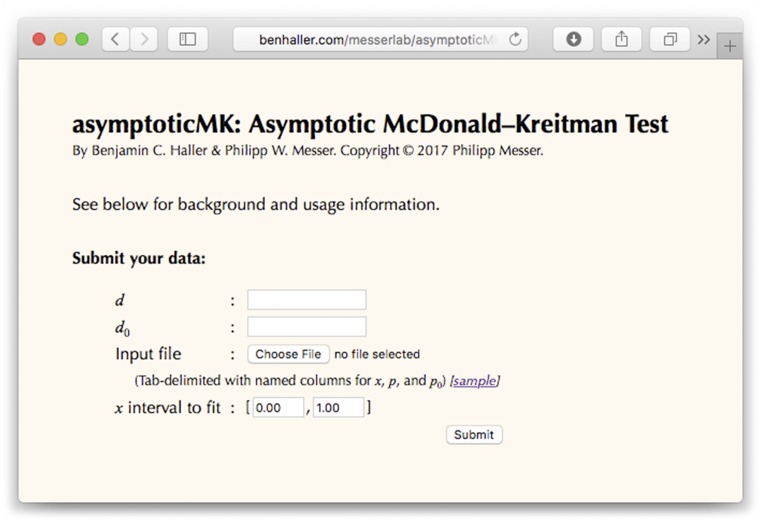
A screenshot of the web page for asymptoticMK. After entering values for *d* and *d*_0_, choosing an input file with binned values for *x*, *p*, and *p*_0_, and choosing the *x* interval to fit, the user can click the Submit button and asymptoticMK will provide its results in a new browser window or tab.

The web service is free to use, without license restrictions of any kind, and is available at http://benhaller.com/messerlab/asymptoticMK.html. That URL displays an entry page (Figure 1) with an input form in which the user may enter the necessary data for the test: *d* (the substitution rate in the test region), *d*_0_ (the substitution rate in the neutral reference region), and an uploaded file containing tab-delimited rows of data with values for *x* (the derived allele frequency), *p*(*x*) (the polymorphism level in the test region at that frequency), and *p*_0_(*x*) (the polymorphism level in the neutral reference region at that frequency). A sample polymorphism file is provided on the website. In practice, it is often advisable to combine polymorphism levels into a smaller number of frequency bins, where *x* then specifies the central frequency of the bin. This is particularly relevant when the data includes frequencies at which no polymorphisms are actually present in the neutral region, in which case *α*(*x*) would be undefined for those particular frequencies according to Equation (2). The frequency bins supplied to asymptoticMK do not need to be equally spaced, but to obtain the best possible *α* estimate it is preferable to have bins providing good coverage across the full frequency spectrum. The input form also allows entry of minimum and maximum values defining a cutoff interval for *x*, such that the test is run using only the polymorphisms whose frequencies fall within that cutoff interval; this is usually desirable as a means of excluding the lowest- and highest-frequency polymorphisms, where SNP quality issues and polarization errors are generally most pronounced. This frequency cutoff is set to [0.1, 0.9] by default, but should be adjusted as needed.

Upon submission of the web form, asymptoticMK conducts its analysis and then opens a results page in a new browser tab, presenting a summary of the input data and the results from the analysis. The first plot on this results page shows binned polymorphism counts, *p*_0_(*x*) and *p*(*x*), for the submitted data; the second plot shows that same data normalized (*i.e.*, the normalized SFS in the test and reference regions). A third plot shows the calculated empirical *α*(*x*) as a function of *x*, estimated from the input data according to Equation (2). The fourth plot shows the same *α*(*x*) data, with the best-fitting model and the asymptotic estimate of *α* from Equation (3) superimposed upon the data.

Below these plots, the results of the analysis are presented in two tables. The first table provides the coefficients *a*, *b*, and (for exponential fits) *c* of the model yielding the best fit to the data. The second table provides the estimated *α*_asymptotic_ according to Equation (3), and the upper and lower limits of the 95% C.I. around that estimate, as well as the estimated *α* from the original nonasymptotic MK test (*α*_original_) for comparison (also estimated from all polymorphisms falling within the frequency cutoff interval specified on the input page).

For purposes of automation, the asymptoticMK web service can also be run at the command line using the Linux/Unix curl command. For example, the command

curl -F”d = 593” -F”d0 = 930” -F”xlow = 0.1” -F”xhigh = 0.9” -F”datafile=@polymorphisms.txt” -F”reply = table” -o “MK_table.txt” http://benhaller.com/cgi-bin/R/asymptoticMK_run.html

would run asymptoticMK with the given values of *d* and *d*_0_, the given *x* cutoff interval, and polymorphism data uploaded from the local file polymorphisms.txt, and would output a simple table of results to the file MK_table.txt. Further documentation on the use of this feature is provided on the asymptoticMK web page.

Finally, it is also possible to run asymptoticMK in R on the user’s local machine. The R code for doing so can be found on asymptoticMK’s GitHub repository. In addition to allowing the user to modify asymptoticMK’s analysis as desired, this option also allows PDF plots to be created, rather than the PNG plots provided by the web-based service.

### Fitting and analysis procedure

The asymptotic MK test first involves calculating values of *α*(*x*) by applying Equation (2) to each frequency bin provided, as described by Messer and Petrov (2013). The next step involves fitting a function *α*_fit_(*x*) to these empirical *α*(*x*) values. For greater robustness, asymptoticMK fits two functions to the data. The first function is exponential, of the form *α*_fit_(*x*) = *a* + *b* exp(−*cx*), and is fitted using the nls2() function, from the R package nls2 (Grothendieck 2013). This fit is done in two steps. First, a brute-force scan for the closest fit is conducted across the likely portion of the three-dimensional parameter space defined by *a*, *b*, and *c*, by exhaustive search. This supplies reasonably good starting values for the second step, which refines those starting values using standard nonlinear least-squares regression. While this two-step procedure generally works well, it can fail to converge if the data are not exponential in form.

To address this possibility of nonconvergence of the exponential fit, asymptoticMK also fits a linear function of the form *α*_fit_(*x*) = *a* + *bx*, with the lm() function that is part of the stats package included in R. This fit always converges, and thus provides a backstop that allows the test to complete even when given irregular or extremely noisy data; however, it is always recommended that the results of the analysis be inspected visually to confirm that they are in fact meaningful.

Once these two models have been fitted, asymptoticMK chooses which model will be used for the remainder of the analysis. If the exponential fit failed to converge, then the linear model is chosen; if both fits succeeded, then the better model is chosen using the Akaike Information Criterion (AIC). Occasionally, in pathological cases, the exponential fit will have the better AIC but will have extremely large coefficient SEs; in this case, the linear fit is chosen since predictions from the exponential model would be effectively worthless.

The chosen model is then used to provide an estimate of the value of *α*_asymptotic_ according to Equation (3), by evaluating the fitted function *α*_fit_(*x*) at *x* = 1; this is the primary result of the test, and provides the test’s estimate of the true value of *α* within the test region. A 95% C.I. around this estimate is also calculated. For the exponential model, this is done using Monte Carlo simulation based upon the fitted model, using the predictNLS() function published online by Spiess (2013); for the linear model, it is done using the standard R function predict().

### Test data sets

To provide a test of asymptoticMK using empirical data, we used the same *Drosophila melanogaster* data set that Messer and Petrov (2013) used in their Figure 3C. This data set consists of SNPs obtained from the genome sequences of 162 inbred fly lines generated by the *Drosophila* genetic reference panel (Mackay *et al.* 2012). Divergence data were obtained from genome alignments between *D. melanogaster* and *D. simulans*, extracted from the 12 *Drosophila* genomes data (Clark *et al.* 2007). The test data in the asymptoticMK analysis (*d* and *p*) are genome-wide nonsynonymous mutations, while synonymous sites were used as the neutral reference (*d*_0_ and *p*_0_). The polymorphism data are available online at asymptoticMK’s GitHub repository, with associated values *d* = 59570 and *d*_0_ = 159058. The default frequency cutoff interval of [0.1, 0.9] was used in the analysis of this data set with asymptoticMK.

We also tested asymptoticMK on simulated data, using the forward genetic simulation framework SLiM 2 (Haller and Messer 2017). A population of 1000 diploid individuals was simulated to evolve in a total of 13 different scenarios, with 20 replicates for each scenario. Simulation runs depended upon six free parameters (*T*, *L*, *μ*, *r*_b_, *s*_d_, and *s*_b_) as described hereafter. After an initial burn-in period of 10,000 generations to equilibrate the model, runs executed for *T* additional generations. The simulated chromosome was *L* base pairs long. Nucleotide mutations occurred uniformly at a rate of *μ* per base per generation, and recombination occurred uniformly at a rate of 10^−7^ per base per generation. Each new mutation was either of neutral type “m1” (relative proportion of 0.5 of all new mutations), of functional nonbeneficial type “m2” (relative proportion of 0.5 of all new mutations), or of functional beneficial type “m3” (relative proportion of *r*_b_ of all new mutations); these relative proportions were automatically rescaled by SLiM to be absolute proportions. The neutral m1 mutations always had a selection coefficient of *s* = 0.0; the selection coefficients of m2 mutations were drawn from a gamma distribution with a mean of *s*_d_ and a shape parameter of 0.2; and m3 mutations always had a selection coefficient of *s*_b_. Fitness effects were assumed to be codominant. Every 500 generations after the burn-in period, all polymorphisms were recorded in the population by dividing them according to their frequency into 50 equal-width frequency bins, and then adding them to an ongoing binned tabulation. The SLiM configuration script used for these simulations is provided on asymptoticMK’s GitHub repository.

The “baseline” parameterization of this model utilized parameter values of *L* = 10^7^, *μ* = 10^−9^, *r*_b_ = 0.0005, *s*_b_ = 0.1, *s*_d_ = −0.02, and *T* = 2 × 10^5^. The other 12 parameterizations involved either a “high” or a “low” value of one of the six parameters, replacing the “central” value used in the baseline scenario: *L* = 10^8^ or 10^6^, *μ* = 10^−8^ or 10^−10^, *r*_b_ = 0.001 or 0.0001, *s*_b_ = 0.2 or 0.02, *s*_d_ = −0.2 or −0.002, and *T* = 2 × 10^6^ or 2 × 10^4^. At the end of each model run, we obtained binned values for *p*(*x*) and *p*_0_(*x*), where *p*_0_ was estimated from all polymorphisms involving mutations of type m1, while *p* was estimated from the combined mutations of types m2 and m3. Values for *d* and *d*_0_ were obtained from the set of mutations fixed during the simulation; as with *p*_0_ and *p*, *d*_0_ was estimated from all mutations of type m1, while *d* was estimated from the combined mutations of types m2 and m3. These values, output by the model, were used in asymptoticMK with the default *x* cutoff interval of [0.1, 0.9] to calculate an *α* estimate. The *α* estimate from the original MK test was also calculated using the data within the same interval. Finally, the true value of *α* was estimated from the simulation run as the fraction *d*_3_ / (*d*_2_ + *d*_3_), where *d*_2_ is the number of m2 mutations fixed and *d*_3_ is the number of m3 mutations fixed. This value provides a metric for the accuracy of the *α* estimates– a benefit of using simulated data, where the true *α* can be calculated.

From this raw data provided by each set of 20 replicates for a given parameterization, summary statistics for that parameterization were computed. In particular, we calculated (i) the mean and SD of the true *α* values, (ii) the mean and SD of the asymptoticMK *α* estimates, (iii) the mean of the absolute differences between the true *α* and the asymptoticMK estimate (*i.e.*, the mean estimation error for asymptoticMK), (iv) the mean and SD of the estimates of *α* using the original nonasymptotic MK test, (v) the mean of the absolute differences between the true *α* and the original MK test estimate (*i.e.*, the mean estimation error for the original MK test), and (vi) the fraction of the 20 replicates for which asymptoticMK chose an exponential (as opposed to linear) fit (Table 1).

**Table 1:** Results from asymptoticMK for simulation runs conducted with SLiM 2

Model	*α*_true_	*α*_asymptotic_	*α*_original_	Δ_asymptotic_	Δ_original_	*ρ*_exp_
Baseline	0.329 ± 0.015	0.307 ± 0.058	0.164 ± 0.035	0.045	0.165	0.75
*L* = 10^8^	0.327 ± 0.008	0.301 ± 0.013	0.174 ± 0.012	0.025	0.152	1.00
*L* = 10^6^	0.321 ± 0.067	0.246 ± 0.134	0.142 ± 0.141	0.120	0.191	0.15
*μ* = 10^−8^	0.306 ± 0.005	0.287 ± 0.016	0.173 ± 0.009	0.019	0.132	1.00
*μ* = 10^−10^	0.317 ± 0.057	0.288 ± 0.169	0.145 ± 0.074	0.134	0.173	0.05
*r*_b_ = 0.0010	0.493 ± 0.018	0.481 ± 0.045	0.378 ± 0.025	0.041	0.114	0.70
*r*_b_ = 0.0001	0.091 ± 0.014	0.115 ± 0.080	−0.103 ± 0.053	0.071	0.194	0.55
*s*_b_ = 0.20	0.477 ± 0.016	0.451 ± 0.032	0.366 ± 0.025	0.029	0.111	0.70
*s*_b_ = 0.02	0.096 ± 0.011	0.090 ± 0.068	−0.119 ± 0.047	0.057	0.215	0.50
*s*_d_ = −0.200	0.424 ± 0.024	0.422 ± 0.042	0.289 ± 0.036	0.032	0.135	0.60
*s*_d_ = −0.002	0.233 ± 0.011	0.234 ± 0.057	0.104 ± 0.039	0.045	0.129	0.50
*T* = 2 × 10^6^	0.324 ± 0.006	0.302 ± 0.014	0.173 ± 0.012	0.022	0.151	1.00
*T* = 2 × 10^4^	0.345 ± 0.063	0.369 ± 0.183	0.225 ± 0.113	0.126	0.120	0.05

The first row shows the averaged results from 20 replicate runs of the baseline SLiM model supplied on GitHub (see text). These runs used parameter values of mutation rate *μ* = 10^−9^ per base position per generation, chromosome length *L* = 10^7^ base positions, beneficial mutation rate *r*_b_ = 0.0005, beneficial mutation selection coefficient *s*_b_ = 0.1, deleterious mutation selection coefficient *s*_d_ = −0.02, and time after burn-in *T* = 2 × 10^5^ generations. Each subsequent row shows the results from 20 replicate runs using the nonbaseline parameter value shown. *α*_true_ specifies the true value of *α* averaged across the 20 replicates in each row; *α*_asymptotic_ and *α*_original_ specify the asymptoticMK estimate and the estimate from the original test, respectively. SDs across the 20 replicates of each row are shown as ± values. Δ_asymptotic_ = |*α*_asymptotic_ − *α*_true_| and Δ_original_ = |*α*_original_ − *α*_true_| specify the mean absolute errors between true *α* values and the estimates from asymptoticMK and the original test, respectively, in each run, averaged over the 20 replicates. *ρ*_exp_ specifies the fraction of runs in which the exponential fit was chosen.

### Data availability

The asymptoticMK web service can be used online at http://benhaller.com/messerlab/asymptoticMK.html. The R source code for asymptoticMK, the SLiM model for the simulations conducted, the *Drosophila* data set analyzed, and other related files are available on GitHub at https://github.com/MesserLab/asymptoticMK. 

## Results and Discussion

**Figure 2:**
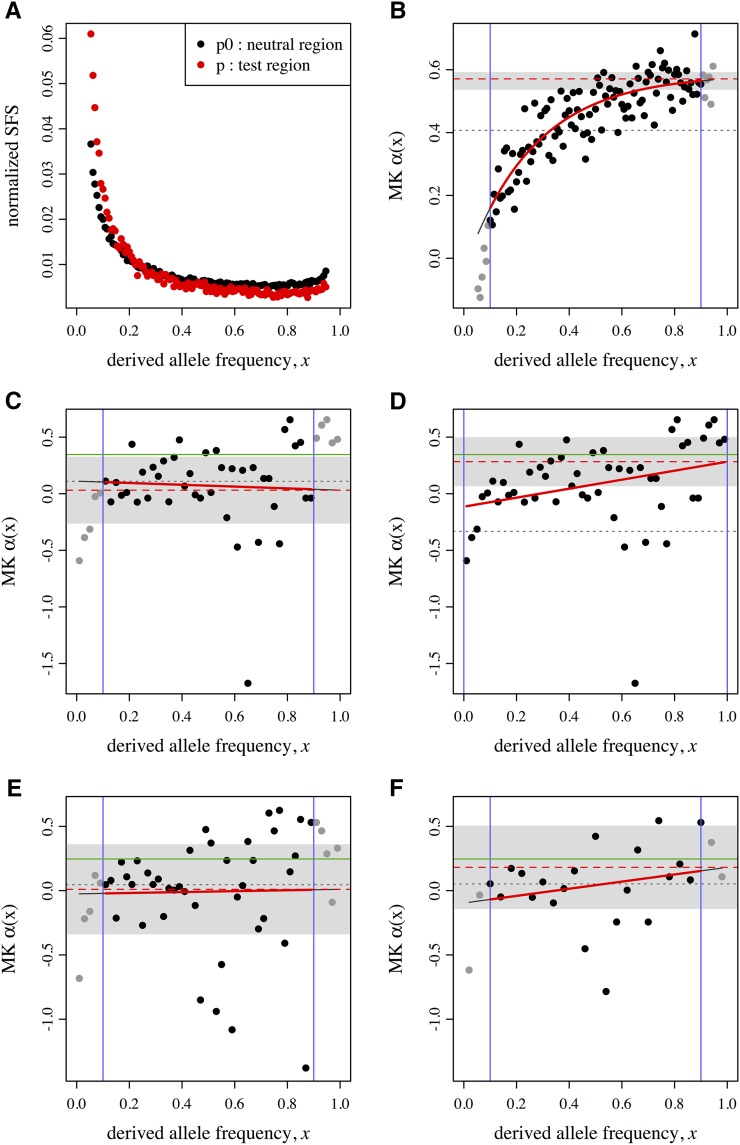
Results from asymptoticMK for three test data sets. (A) Normalized site frequency spectrum (SFS) for the *Drosophila* data set used in Messer and Petrov (2013). Points show normalized binned polymorphism frequencies for the neutral region (black) and the test region (red). (B) Result of asymptoticMK’s analysis of that data set. The two vertical blue lines show the limits of the frequency cutoff interval used for fitting. Points indicate binned values of *α*(*x*), estimated according to Equation 2; points are gray if they are outside the cutoff interval (and thus not used in fitting). The solid red curve shows the fitted *α*_fit_(*x*) (here, exponential). The dashed red line shows the estimate of *α*_asymptotic_, obtained from the fitted function according to Equation 3. The gray band indicates the 95% C.I. around this *α*_asymptotic_ estimate. The dotted gray line shows the estimate of *α*_original_, obtained from the original (nonasymptotic) McDonald–Kreitman (MK) test, for comparison (also calculated using only the data within the cutoff interval). (C) and (D) show corresponding results from one SLiM simulation run, and (E) and (F) show results from another SLiM simulation run; in each case, the first panel shows the result of an automated fit using asymptoticMK, whereas the second shows the improvement after hand tailoring of the fit (see *Results and Discussion*). Note that in all four cases, the linear fit was deemed more appropriate by asymptoticMK. The solid green horizontal lines, finally, show the true value of *α* in the simulation runs for comparison.

Results from our test of asymptoticMK with the empirical *D. melanogaster* data set are shown in Figure 2, A and B. The fitted exponential function is: *α*_fit_(*x*) = 0.585 − 0.622 exp(−3.80*x*). The asymptotic MK estimate provided by this model is *α*_asymptotic_ = 0.571. These results match those obtained by Messer and Petrov (2013) using the same data set (their Figure 3C), as expected. The estimate provided by the original MK test is *α*_original_ = 0.407, by comparison (shown in Figure 2B).

The results from the analysis of the SLiM simulations are shown in Table 1. In 12 of the 13 parameterizations, the mean estimation error of asymptoticMK was markedly lower than that of the original MK test; in the other parameterization (*T* = 2 × 10^4^) the tests performed similarly (mean estimation errors of 0.126 and 0.120). For three of the 13 parameterizations (*L* = 10^6^, *μ* = 10^−10^, and *T* = 2 × 10^4^), however, the mean estimation error of asymptoticMK was > 0.1, indicating that *α* estimates were relatively inaccurate for those simulations. These three parameterizations involved a shorter chromosome, a lower mutation rate, or a shorter duration, and thus all provided ∼10 times less polymorphism data upon which to base estimations than our baseline scenario. Accordingly, parameterizations that provided more polymorphism data (*L* = 10^8^, *μ* = 10^−8^, and *T* = 2 × 10^6^) provided more accurate *α* estimates (mean estimation errors below 0.03). This pattern was weak or absent for the original MK test; even for the high-data parameterizations the original MK test always showed a mean estimation error > 0.1, and its mean estimation error for the high-data *L* = 10^8^ case was actually substantially higher than for the low-data *L* = 10^6^ case (0.151 *vs.* 0.120). This is consistent with the fact that the original MK test systematically underestimates *α* in the presence of deleterious mutations (as discussed in the opening paragraphs). The asymptotic MK test may still have a tendency toward underestimation as well, but errors are much smaller.

Another noteworthy observation is that in the high-data parameterizations the exponential fit was chosen by asymptoticMK in 100% of cases, whereas in the low-data parameterizations the linear fit was chosen a majority of the time (Table 1). It is not the case that the linear model always produces a poor *α* estimate; we observed many runs where the linear fit performed well. However, it may indicate that a poor cutoff interval was chosen, that the binning of the polymorphism data ought to be done differently, or that the data are simply too noisy. We suggest that the result of the asymptotic MK test should always be inspected visually to verify that the fit is reasonable and that appropriate cutoff intervals and bin sizes were used.

To illustrate how such manual inspection can help improve estimates, we examine two of the simulation runs from Table 1 in more detail. Figure 2C shows the automated fit for one of the simulations in the low-data *L* = 10^6^ scenario. A linear fit function produced an asymptotic *α* estimate of 0.0313, which is quite distant from the true value of 0.3462. The binned polymorphism data within the cutoff interval of [0.1, 0.9] is rather flat, but the data appears reasonable across the whole frequency spectrum in this case, and the upward trend of the data is much more visible outside of the cutoff interval used. Changing the cutoff interval to [0.0, 1.0] produces the fit shown in Figure 2D with an asymptotic *α* estimate of 0.2829, much closer to the true value. Figure 2E shows the automated fit for another *L* = 10^6^ scenario run. This fit also used a linear fit function, producing an asymptotic *α* estimate of 0.0103 compared to the true value of 0.2462. Here, the data are very noisy, which could be an indication that more bins have been used than can be robustly supported by the data. Rebinning the polymorphism data into half as many bins provides a less noisy data set that results in a much better fit (Figure 2F), with an *α* estimate of 0.1813– again, a substantial improvement. These examples illustrate that automated fits can be particularly problematic in low-data situations such as the *L* = 10^6^ scenario, but that hand inspection and tailoring of the fitting process can sometimes improve the result noticeably.

### Conclusions

In this paper, we presented asymptoticMK, a new web-based tool for executing the asymptotic MK test. To demonstrate its functionality, we analyzed both empirical and simulation-generated data sets. Our results illustrate the greater power of the asymptotic MK test to estimate the true value of *α*, compared to the original nonasymptotic test. However, our results also underline the need for a large data set to obtain reasonably accurate results from the asymptotic test; estimates of *α* from a single gene, or from a system with a very short divergence time, are unlikely to be meaningful. In addition, visual inspection of the quality of the fit used to estimate *α* is necessary for accuracy. With attention to these caveats, the asymptoticMK service presented here allows the user to obtain *α* estimates quickly and easily through any web browser, or using R on any machine.
